# Sex differences in mild vascular cognitive impairment: A multimodal transcranial magnetic stimulation study

**DOI:** 10.1371/journal.pone.0282751

**Published:** 2023-03-03

**Authors:** Mariagiovanna Cantone, Francesco Fisicaro, Raffaele Ferri, Rita Bella, Giovanni Pennisi, Giuseppe Lanza, Manuela Pennisi

**Affiliations:** 1 Neurology Unit, University Hospital Policlinico “G. Rodolico-San Marco”, Catania, Italy; 2 Department of Biomedical and Biotechnological Sciences, University of Catania, Catania, Italy; 3 Clinical Neurophysiology Research Unit, Oasi Research Institute-IRCCS, Troina, Italy; 4 Department of Medical and Surgical Sciences and Advanced Technologies, University of Catania, Catania, Italy; 5 Department of Surgery and Medical-Surgical Specialties, University of Catania, Catania, Italy; Universita degli Studi di Trento, ITALY

## Abstract

**Background:**

Sex differences in vascular cognitive impairment (VCI) at risk for future dementia are still debatable. Transcranial magnetic stimulation (TMS) is used to evaluate cortical excitability and the underlying transmission pathways, although a direct comparison between males and females with mild VCI is lacking.

**Methods:**

Sixty patients (33 females) underwent clinical, psychopathological, functional, and TMS assessment. Measures of interest consisted of: resting motor threshold, latency of motor evoked potentials (MEPs), contralateral silent period, amplitude ratio, central motor conduction time (CMCT), including the F wave technique (CMCT-F), short-interval intracortical inhibition (SICI), intracortical facilitation, and short-latency afferent inhibition, at different interstimulus intervals (ISIs).

**Results:**

Males and females were comparable for age, education, vascular burden, and neuropsychiatric symptoms. Males scored worse at global cognitive tests, executive functioning, and independence scales. MEP latency was significantly longer in males, from both sides, as well CMCT and CMCT-F from the left hemisphere; a lower SICI at ISI of 3 ms from the right hemisphere was also found. After correction for demographic and anthropometric features, the effect of sex remained statistically significant for MEP latency, bilaterally, and for CMCT-F and SICI. The presence of diabetes, MEP latency bilaterally, and both CMCT and CMCT-F from the right hemisphere inversely correlated with executive functioning, whereas TMS did not correlate with vascular burden.

**Conclusions:**

We confirm the worse cognitive profile and functional status of males with mild VCI compared to females and first highlight sex-specific changes in intracortical and cortico-spinal excitability to multimodal TMS in this population. This points to some TMS measures as potential markers of cognitive impairment, as well as targets for new drugs and neuromodulation therapies.

## Introduction

### Background

Vascular cognitive impairment (VCI) is a group of cognitive disorders resulting from cerebrovascular pathology [1]. VCI is the second most common dementia, and ~50% of those with Alzheimer’s disease (AD) have co-morbid VCI pathology [2]. The severity of VCI runs along a wide spectrum, ranging from milder forms (mild VCI) to severe vascular dementia (VaD). Symptoms of VCI vary, mostly depending on insult location and burden, although they often include mental slowness, executive dysfunction, and memory loss, along with a variety of motor, behavioral, and psychological disturbances (depression and apathy) [3].

VCI pathogenesis typically begins with cerebral small vessel disease, carotid artery occlusion/stenosis, or stroke, but the small vessel pathology remains the most common cause, resulting in a subcortical ischemic vascular disease [4]. Other vascular risk factors, such as cardiovascular diseases, hypertension, and diabetes, can also contribute, eventually leading to or worsening cerebral hypoperfusion [5]. In addition to cognitive tests, VCI diagnosis is dependent on neuroimaging and, therefore, may be under-diagnosed or even undiagnosed in the absence of stroke [6]. However, VCI is clearly visualized on brain magnetic resonance imaging (MRI) and may include: infarcts or microinfarcts, small vessel disease, hemorrhages or microbleeds, and white matter hyperintensities (WMHs) due to ischemia/hypoperfusion [7]. To date, there is no effective treatment for VCI and current pharmacological approaches are only mildly effective for alleviating symptoms in the short-term [8, 9]. Therefore, identifying risk factors and targeting reliable biomarkers would be the most effective mean for fighting VCI [10–12].

Although prevalence of dementia is globally higher in females [13], this sex difference appears to be largely driven by the AD-related dementia [14]. Conversely, VaD may be more common in males, although data is mixed. A few studies show trends for higher risk in males which, however, did not reach statistical significance, while others have observed no difference [15, 16]. Yet, the Rotterdam Study showed that VaD incidence was significantly higher among males in the 75–90-year age groups [17]. Additionally, the Italian Longitudinal Study on Aging (ILSA) study found a significantly higher risk of VaD in males than in females [18]. A possible explanation for these inconclusive results might lie on the age of the cohorts examined. A meta-analysis on European studies found that sex differences in prevalence of VaD reversed their trend with advancing age: before the age of 79, VaD is more prevalent in males, but after the age of 85 it is more prevalent in females [19]. This is in agreement with the Rotterdam Study, which also found this reversal of sex differences in those ≥90 years of age [17]. Nevertheless, caution should be used when interpreting data in very old populations, due to the survival bias of females.

While males appear to be globally at higher risk for VCI, some risk factors more adversely affect women, such as pregnancy-related disorders, menopause, and poorly timed hormone replacement therapy (HRT) [3]. On the contrary, other female-specific factors may provide protection, including healthy pregnancies and HRT soon after menopause (although more evidence is needed before this can be recommended). Furthermore, the presence of co-morbid risk factors, such as diabetes, obesity, and hypertension, may more adversely affect females than males. In contrast, some risk factors more greatly affect males, such as hyperlipidemia and myocardial infarction [3]. Moreover, stroke, one of the leading cause of VCI, has a higher incidence in males than in women throughout much of the lifespan [20].

### Transcranial magnetic stimulation (TMS)

TMS was originally introduced as a non-invasive neurophysiological tool able to evaluate the excitability of the primary motor cortex (M1) and the conductivity along the cortical-spinal tract, thus being applied in a number of cerebral and/or spinal disorders affecting the motor system, even subclinically [21–23]. Today, however, TMS goes well beyond, being used to study the pathophysiology of neurological and neuropsychiatric diseases, to probe *in vivo* and in “real-time” the excitability and plasticity of the human brain, and to assess the functioning of intracortical circuitries and callosal fibers [24, 25]. As such, TMS is well suited for the exploration and monitoring of several disorders, including their early stages [26–29] and some systemic diseases involving the brain [30, 31]. Because TMS can assess the effects of drugs that are agonists or antagonists for specific transmitters, each measure also explores a neurochemical pathway, such as that mediated by glutamate, γ-amino-butyric acid (GABA), or acetylcholine (“pharmaco-TMS”) [32].

TMS is based on the Faraday’s law of electromagnetic induction: a transducing coil, attached to a high-voltage high-current discharge system, produces a strong time-varying and short-lasting magnetic field to the stimulation coil. When the coil is placed tangentially to the head, the magnetic field penetrates the skull with minimal attenuation and induces a secondary current in conductive intracranial tissue. The electrical field in the tissue is oriented perpendicular to the magnetic field and opposite to the direction of the electrical current in the stimulation coil [33]. Motor evoked potentials (MEPs) are produced by stimulating the M1 at the optimum scalp position to elicit motor responses in the contralateral distal target muscle. The operator can control the intensity of the current flowing through the coil [33] and also manipulate frequency and interstimulus interval (ISI) of the delivered stimuli, which critically determine the TMS effects [34].

Single TMS pulses over the M1 elicits a MEP in contralateral muscles. Both MEP latency and central motor conduction time (CMCT) are indexes of integrity of the cortical-spinal tract, whereas the MEP amplitude (i.e., the peak-to-peak size of the motor response) mainly reflects the excitation state of output cells in the motor cortex, nerve roots, and peripheral motor tracts, till the muscle [34]. MEP latency is the time interval between the administration of TMS over the M1 and the MEP onset from the contralateral muscle; as such, it reflects the total conduction time, accounting for both central and peripheral nervous system conductivity, as well as for neuromuscular junctions and muscles; conversely, CMCT, obtained as the latency difference between MEPs induced by stimulation of the motor cortex and that evoked by spinal (motor root) stimulation, reflects the integrity of the cortical-spinal tract only, from upper to lower motor neurons [35].

The resting motor threshold (rMT) is a global parameter of brain excitability, since it is a compound measure of the membrane excitability of cortical-spinal neurons, neural inputs into pyramidal cells within the cortex, and spinal motor neurons, neuromuscular junctions, and muscles. Suprathreshold TMS pulse to the M1 during a tonic voluntary contraction of the contralateral muscles suppresses the electromyographic (EMG) activity in those muscles for a few hundred milliseconds [36]. This phenomenon, called contralateral cortical silent period (CSP), can be exploited to functionally measure some intracortical inhibitory circuits, mainly mediated by GABA-B transmission [37].

Inhibitory and excitatory interneuronal activity within the human cortex can be non-invasively explored by using the paired-pulse TMS paradigm [38, 39]. The conventional protocol uses a “conditioning stimulus” (CS, subthreshold) followed by a “test stimulus” (TS, suprathreshold). By varying the intensity of the CS and the ISI between the pair of TMS pulses, several measures of intracortical interneuronal function and interaction can be obtained. At ISI of 1–5 ms, the CS suppresses the MEP amplitude (i.e., the short-latency intracortical inhibition, SICI) [38], whereas at a longer ISI (7–20 ms), the stimulus results in an enhanced MEP response (i.e., the intracortical facilitation, ICF) [39]. While SICI is likely mediated by the GABA-A interneuron activity [40], ICF is a more complex phenomenon [41], although mainly produced by the activation of glutamatergic neurons [42].

TMS can be also used to test the sensory-motor interaction. For instance, the short-latency afferent inhibition (SAI) of the MEP reflects the sensory stimuli-mediated inhibitory modulation of the M1 [43]. This effect depends on the interval between the peripheral nerve electrical stimulus and the TMS pulse, and it typically occurs at an ISI of 20 ms. SAI mainly represents the neurophysiological correlate of central cholinergic activity, because it is reduced or abolished by the muscarinic receptor antagonist scopolamine and it is positively modulated by acetylcholine [44].

### Aim and hypothesis

In the last 20 years, TMS has been applied in patients with dementia, allowing the identification of potential markers or predictors of cognitive decline. As recently reviewed, TMS provides insights into the pathophysiology, progression, and response to treatment in different forms of dementia, including AD and other degenerative disorders [45], as well as in a number of secondary dementias [46]. Although a single TMS index offers low specificity, the use of a panel of measures can support the clinical diagnosis and possibly predict progression. Moreover, when applied repetitively, TMS holds promise as a therapeutic intervention, although, so far, only repetitive TMS over the left dorsolateral prefrontal cortex and the multisite stimulation associated with cognitive training have been shown to be, respectively, possibly (Level C of evidence) and probably (Level B of evidence) effective to improve cognition, apathy, memory, and language in AD patients, especially at a mild/early stage of the disease [45].

In VCI, TMS has overall revealed enhanced cortical excitability, that seem to correlate with the disease process. In some patients, such pattern may be considered as an adaptive response to disease progression, thus allowing the plasticity of motor programming and execution [26]. Findings also point out the possibility to employ TMS to predict cognitive deterioration in the so-called “brains at risk” for dementia, which may be those patients who benefit more of disease-modifying drugs and rehabilitative or neuromodulatory approaches [26]. Finally, TMS may select the responders to specific drugs in the attempt to maximize the response and to improve maladaptive plasticity [47]. In VCI, while no single TMS index owns enough specificity, a panel of TMS-derived measures can support diagnosis and identify early markers of progression into VaD [45].

To date, however, a direct and multimodal TMS comparison between males and female patients with mild VCI is lacking. Here, we determine any difference in cognition, independence, and TMS between males and females and correlate TMS findings with clinical-cognitive and imaging data. Clinically, the results will assess the relevance of sex for an accurate and meaningful MEPs interpretation in this population. From a research perspective, this will not only provide further insights on VCI pathophysiology, but also point to some TMS measures as potential markers of cognitive impairment and targets for new drugs and neuromodulation therapies. Accordingly, we hypothesized that, since pathophysiology may differ between sexes in VCI, this will be reflected in differences also at clinical level and TMS measurements.

## Materials and methods

### Subjects and assessment

This was a cross-sectional study that consecutively recruited all elderly patients (≥65 years) satisfying the clinical criteria for mild VCI [1]. All subjects attended the outpatient Cerebrovascular Center of the Azienda Ospedaliero-Universitaria Policlinico “G. Rodolico-San Marco” of Catania, Italy. According to the latest Classification Consensus Study of the Vascular Impairment of Cognition [48], mild VCI identifies a clinically and radiologically homogeneous group of non-demented patients with small vessel disease (lacunar infarcts and/or ischemic WMHs), primarily located subcortically. Based on these criteria, 60 right-handed patients (27 males, 33 females; mean age ± standard deviation 70.5 ± 6.3 and 66.8 ± 5.4 years, respectively) were recruited.

According to the International Society for Vascular Behavioral and Cognitive Disorders [49], all the VCI patients included showed impairment in at least one cognitive domain and normal activities of daily living [48, 49]. As such, none of them met the criteria for dementia of the Diagnostic and Statistical Manual of Mental Disorders, Fifth Edition [50], whereas they all fulfilled the MRI criteria for subcortical ischemic vascular disease with WMHs [51].

Patients were treated for their vascular risk factors with antiplatelet or anticoagulant medications (aspirin, clopidogrel, warfarin), anti-hypertensive drugs (angiotensin-converting enzyme inhibitors, angiotensin II receptor antagonist, diuretics, dihydropyridine calcium channel blockers), cholesterol lowering medications (statins), and oral antidiabetic drugs or insulin. None of them was on antidepressant treatment, psychotropic medicament, or any other drug potentially affecting TMS.

Exclusion criteria were: Mini-Mental State Examination (MMSE) score < 24 [52]; history of stroke/transient ischemic attack or other neurological disorders (e.g., Parkinson’s disease, Multiple Sclerosis, traumatic brain injury, epilepsy); major psychiatric disorders (e.g., schizophrenia, obsessive-compulsive disorder, major depressive disorder); family or personal history of early-onset major depression; any severe acute or chronic medical illness; alcohol or drug abuse; any condition precluding MRI or TMS execution.

Clinical and demographic assessment included: age, sex, education, cardio- and cerebrovascular risk factors, personal or family history of depression, neurological signs and symptoms. The right-handedness of all participants was assessed by the Edinburgh Handedness Inventory [53].

All subjects underwent a psycho-cognitive battery of tests, including: global screening tools of cognitive impairment, i.e., MMSE and Montreal Cognitive Assessment Test (MoCA) [54]; evaluation of executive functions through the Frontal Assessment Battery (FAB) and the Stroop Color-Word Test interference (normative values collected from an Italian population sample: Stroop T score, for the time needed to complete the task; Stroop E: number of errors during the task execution [55]); assessment of the functional status by means of the Activity of Daily Living (ADL) and the Instrumental Activity of Daily Living (IADL); quantification of depressive symptoms and apathy by means of the Hamilton Depression Rating Scale (HDRS) [56] and the Apathy Evaluation Scale (AES) [57], respectively, both of which have proved to be a valid tool in VCI [58, 59]. All analyses on cognitive performance were adjusted for age and education. The battery was performed by a trained neurologist (M.C.) expert in psychometric evaluation.

MRI was acquired using a 1.5-T General Electric system. The protocol included T1-, T2-, proton density-weighted, and fluid-attenuated inversion recovery scans; the slice thickness was 5 mm, with a 0.5 mm slice gap. The severity of WMHs was graded according to the visual scale score of Fazekas: 0 = absence; 1 = punctuate foci; 2 = partially confluent foci; 3 = large confluent areas [60].

This study was carried out in accordance with the Declaration of Helsinki of 1964 and its later amendments. The protocol was approved by the Ethics Committee of the Azienda Ospedaliero-Universitaria Policlinico “G. Rodolico-San Marco” of Catania, Italy (protocol code: 292/prot. n. 871), and all subjects gave written informed consent prior to entry.

### TMS procedures

TMS was performed using a high-power Magstim 200 mono pulse monophasic magnetic stimulator (Magstim Co., Whitland, Dyfed, UK). A 70 mm figure-of-eight coil was held tangentially over the motor cortex at the optimum scalp position to elicit MEPs in the contralateral first dorsal interosseous (FDI) muscle. EMG activity was recorded from a silver/silver chloride surface active electrode placed over the motor point of the target muscle, with the reference electrode placed distally at the metacarpal-phalangeal joint of the index finger. Motor responses were amplified and filtered (bandwidth: 3–3,000 Hz) using a 2-channel Medelec Synergy system (Oxford Instruments Medical, Inc., UK), with an amplification factor of the screen of 100 *μ*V per division unit for the measurement of rMT and 1 mV per division unit during MEP recording. The temporal resolution of the screen was 5 ms per division unit, in such a way that the TMS artifact, the beginning, and the end of each MEP were always visible [34].

For the motor nerve conduction study, a bipolar nerve stimulation electrode, with 6 mm diameter felt pads and an interelectrode separation of 25 mm, was used. M and F waves were elicited by giving supramaximal electrical stimulation (constant current square wave pulse of 0.2 ms) to the ulnar nerve at wrist, bilaterally. Three reproducible artifact-free M responses and ten F waves were recorded for each side of the subjects. While FDI muscle was relaxed, the peak-to-peak amplitude of M and F waves was determined. The F wave with the shortest latency, providing a measure of conduction in the fastest axons [34], was considered.

Measures of motor cortex excitability included: rMT, CSP, MEPs, amplitude ratio (A ratio), and CMCT, from both hemispheres. Resting MT was defined as the lowest stimulus intensity able to elicit MEPs of an amplitude >50 *μ*V in at least 5 of 10 trials, with the muscle at rest [34]. The CSP was determined with an approximately 50% of maximum tonic voluntary contraction of the FDI muscle, induced by contralateral TMS pulses delivered at 130% of the rMT. During the CSP recording, the subjects maintained the isometric tonic contraction by abducting the index finger against a strain gauge. Mean CSP duration, based on trial-by-trial measurements of 10 rectified traces, was calculated. Following the latest guidelines of the International Federation of Clinical Neurophysiology [34], single trial CSP was measured as the time elapsing from the MEP onset until the recurrence of voluntary tonic EMG activity. MEP size was measured as a percentage of the supramaximal M size (A ratio), which is more reliable than the peak-to-peak MEP size [34].

CMCT was calculated by subtracting the conduction time in peripheral nerves from MEP latency obtained during moderate active muscle contraction (10–20% of maximum background force), at a stimulus intensity set at 130% of the rMT [34]. By using the F wave latency, CMCT-F (ms) was estimated as: T − (F + M − 1) / 2 [T = onset latency of MEP elicited by TMS; F = onset latency of the F wave by electrical ulnar nerve stimulation; M = onset latency of the M wave by electrical ulnar nerve stimulation]. Moreover, in order to assess spinal motor excitability, the mean amplitude of the F wave was measured [34].

Intracortical circuits were studied bilaterally using the CS-TS paradigm [38] through a BiStim module (Magstim Co., Whitland, Dyfed, UK) connected to a Cambridge Electronic Design (CED) Micro 1401 Interface (Cambridge, UK). The procedure consisted of applying two magnetic stimuli in rapid succession through two magnetic stimulators connected to each other. CS was applied at 80% of the subject’s rMT, whereas TS was set at 130% of the rMT. ISIs tested were 1, 3, and 5 ms for SICI and 7, 10, and 15 ms for ICF. Ten trials for each ISI were recorded randomly and an algorithm, whose properties approximate the sequence of random numbers (pseudorandom number generator), was used. The responses were expressed as the ratio of the MEP amplitude produced by paired stimulation to that produced by TS alone, as recommended [34].

SAI was studied using a high-voltage Digitimer Stimulator, model DS7A (Digitimer Ltd, Welwyn Garden City, UK) [43]. Peripheral CS consisted of single pulses of electrical stimulation (200 μs duration) applied through bipolar electrodes to the right median nerve at wrist (cathode proximal). Paired stimulation was obtained with a 70-mm figure-of-eight coil through the BiStim module connected to the CED Micro 1401, interface allowing both stimulus generation and data capture. The intensity of the peripheral nerve CS was set just above the motor threshold necessary to evoke a visible twitch of the thenar muscles [34]. The afferent inhibition induced by the peripheral CS was tested at different ISIs, based on the latency of the N20 component of the somatosensory evoked potentials (SEPs) obtained from the left hemisphere after stimulation of the right median nerve. To record SEPs, the active electrode was placed 3 cm posterior to C3 (according to the 10–20 international electroencephalography system) and the reference electrode on the forehead. A total of 500 responses were obtained and averaged from two different trials (250 each) to identify the optimal latency of the N20 peak. ISIs at the latency of N20 plus 2 ms (N20+2) and N20 plus 8 ms (N20+8) were investigated, given that at these intervals it is known to occur a clear inhibition of the cortical–spinal volleys evoked by TMS [43]. Ten repeats were delivered for cortical stimulation alone, without electrical peripheral CS (unconditioned MEP) and for CS at each ISI, in a pseudo-randomized order and with an inter-trial interval of 10 s, as recommended [34]. SAI was measured as the amplitude ratio between conditioned (N20+2; N20+8) and unconditioned MEP response, expressed in percentage (N20+2 ratio, %; N20+8 ratio, %).

A standardized safety checklist screened all individuals before TMS execution [61] and to exclude any contraindication or medication affecting the cortical excitation state. All procedures were performed with participants seated in a dedicated armchair with constant EMG monitoring to guarantee a desirable level of tonic muscle activity during contraction or a total muscle relaxation. Once collected, data were stored on a dedicated PC by means of an *ad hoc* software that allows to acquire, process, and analyze data [62]. To reduce the interindividual variability, TMS recordings were performed in the same lab and experimental conditions, at the same time of the day (10:30–11:30 a.m.), and by the same trained operators (F.F. and G.L.).

### Statistical analysis

Subjects’ characteristics were handled as means and standard deviations (continuous variables) or frequencies (categorical variables). Continuous variables obtained from males and females were compared by means of the Student’s *t*-test accompanied by the calculation of the Cohen’s *d* effect size, defined as the difference between two means divided by the pooled standard deviation for those means. According to Cohen, 0.2 is indicative of a small effect, 0.5 of a medium and 0.8 of a large effect size [63]. The comparison of categorical variables obtained from the same groups of subjects was done by means of the *χ*^2^ or the Fisher exact test, as appropriate.

Then, based on the results, we checked for any simultaneous association of demographic (age, sex) and anthropometric features (height, weight) or neurocognitive test scores (predictors) on TMS findings (dependent variables) by means of the General Regression Models of the software STATISTICA v.6 StatSoft Inc. This module allows to build models for design with categorical predictor variables, as well as with continuous predictor variables.

For each dependent variable, the statistical significance of the association of sex was obtained by considering the effect of the other independent predictor factors. Finally, we analyzed the association between clinical-cognitive and TMS data by calculating the Pearson’s correlation coefficient and, following the Cohen’s indications [63], we considered a correlation coefficient 0.10, 0.30, and 0.50 as corresponding to small, medium, and large sizes, respectively. P value was considered as statistically significant when <0.05.

## Results

All subjects completed the whole procedure, and no adverse event or undesirable effect was reported. None of the patients had focal neurological deficit. Clinical-demographic and neuropsychological characteristics of participants are summarized in Table 1. The two groups of patients were comparable in terms of age, education, and sex distribution. The number and type of vascular risk factors, differed by sex, were equally distributed, except for diabetes, which was significantly more reported in males than in females. The frequency and severity of WMHs, according to the Fazekas visual scale, as well as symptoms of depression and apathy, were also comparable between sexes. As expected, males’ height and weight were significantly higher than females. Cognitively, male participants scored significantly worse at MMSE, MoCA, FAB, ADL, and IADL; conversely, Stroop performance (T and E scores), HDRS, and AES did not differ between sexes.

**Table 1:** Demographic, anthropometric, and psycho-cognitive features of male and female patients.

	**Females**		**Males**		***t*-value**	***p***	**Effect size**
	**Mean**	**SD**	**Mean**	**SD**			**Cohen’s *d***
**Age, years**	66.8	5.4	70.5	6.3	-1.8	NS	-0.631
**Height, cm**	157.1	5.7	163.6	5.6	-3.1	0.004	-1.150
**Weight, Kg**	63.6	9.5	73.5	10.8	-2.7	0.01	-0.973
**Education, years**	7.9	4.7	7.8	4.7	0.0	NS	0.021
**MMSE**	27.3	1.7	25.7	1.3	2.9	0.007	1.057
**MoCA**	23.1	2.1	20.1	2.1	3.9	0.001	1.429
**ADL**	5.9	0.2	5.5	0.7	2.8	0.01	0.777
**IADL**	7.6	0.6	6.6	1.0	3.6	0.001	1.213
**HDRS**	16.8	5.6	14.9	4.4	1.0	NS	0.377
**AES**	1.2	0.6	1.3	0.5	-0.6	NS	-0.181
**Stroop T**	41.8	17.0	44.9	14.8	-0.5	NS	-0.195
**Stroop E**	3.5	2.4	3.2	2.2	0.3	NS	0.130
**FAB**	15.7	1.1	14.5	2.0	2.1	0.04	0.743
	**Females**		**Males**				
	**Yes**	**No**	**Yes**	**No**	**Fisher exact test**		
**Hypertension**	29	4	23	4	NS		
**Atrial Fibrillation**	4	29	2	25	NS		
**CCAD**	1	32	8	19	NS		
					***χ*** ^**2**^		***p***
**Dyslipidemia**	20	13	13	14	0.93		NS
**Smoke habit**	8	25	6	21	0.03		NS
**Diabetes**	4	29	13	14	9.49		0.002
**Clinical signs/symptoms**	15	18	16	11	1.13		NS
**Family history of depression**	12	21	5	22	2.33		NS
**History of depression**	13	20	7	20	1.21		NS
**Fazekas visual scale score**	**F1**	**F2**	**F3**	***χ*** ^**2**^	***p***		
Females	17	12	4	2.94	NS		
Males	12	7	8				

(in alphabetical order) ADL = Activity of Daily Living; AES = Apathy Evaluation Scale; CCAD = chronic coronary artery disease; F = females; FAB = Frontal Assessment Battery; White matter hyperintensities according to the Fazekas visual scale score: 1 = punctuate foci (F1); 2 = partially confluent foci (F2); 3 = large confluent areas (F3); HDRS = Hamilton Depression Rating Scale; IADL = Instrumental Activity of Daily Living; M = males; MMSE = Mini Mental State Examination; MoCA = Montreal Cognitive Assessment Test; NS = not significant; SD = standard deviation; Stroop E = Stroop Color-Word Test interference, number of errors during the task execution; Stroop T = Stroop Color-Word Test interference, time needed to complete the task; bold numbers = statistically significant *p* values.

Regarding TMS (Table 2), MEP latency was significantly longer in males, from both sides, as well CMCT and CMCT-F from the left hemisphere, whereas those calculated from the right side exhibited a similar trend, though not statistically significant. The other single-pulse TMS measures, i.e., rMT, CSP, A ratio, and F wave amplitude, were similar between the two groups. Paired-pulse TMS indexes, i.e., SICI at 1, 3, and 5 ms bilaterally, ICF at 7, 10, and 15 ms bilaterally, and SAI at 2 and 8 ms from the left hemisphere, did not reveal any difference, except for a significant lower SICI at ISI of 3 ms from the right hemisphere in males.

**Table 2:** TMS measures of male and female participants with mild vascular cognitive impairment.

	Females		Males		t-value	*p*	Effect size
	Mean	SD	Mean	SD			Cohen’s *d*
**L rMT, %**	47.6	8.37	44.1	7.52	1.21	NS	0.440
**L CSP, ms**	93.0	40.38	90.9	35.38	0.15	NS	0.055
**L MEP Lat, ms**	18.6	1.15	21.6	2.10	-5.04	0.000025	-1.772
**L CMCT, ms**	5.4	1.07	6.3	0.92	-2.29	0.03	-0.902
**L CMCT-F, ms**	5.0	0.99	6.1	0.92	-3.05	0.005	-1.151
**L Amp ratio**	0.4	0.13	0.3	0.15	2.02	NS	0.712
**L F Amp, mV**	0.2	0.12	0.1	0.06	0.88	NS	1.054
**L SICI, ISI 1 ms**	0.5	0.35	0.6	0.32	-0.70	NS	-0.298
**L SICI, ISI 3 ms**	0.4	0.34	0.6	0.29	-0.87	NS	-0.633
**L SICI, ISI 5 ms**	1.0	0.55	1.0	0.58	0.25	NS	0.000
**L ICF, ISI 7 ms**	1.2	0.55	1.3	0.42	-0.42	NS	-0.204
**L ICF, ISI 10 ms**	1.5	0.57	1.5	0.52	0.15	NS	0.000
**L ICF, ISI 15 ms**	1.5	0.49	1.5	0.54	0.02	NS	0.000
**R rMT, %**	42.9	2.78	42.3	4.07	0.46	NS	0.172
**R CSP, ms**	80.7	35.32	103.7	35.42	-1.70	NS	-0.650
**R MEP Lat, ms**	18.7	1.24	21.2	2.10	-3.93	0.00056	-1.450
**R CMCT, ms**	5.4	1.04	6.1	0.89	-1.94	NS	-0.723
**R CMCT-F, ms**	4.9	1.00	5.7	1.11	-1.95	NS	-0.757
**R Amp ratio**	0.5	0.19	0.4	0.11	0.12	NS	0.644
**R F Amp, mV**	0.1	0.07	0.1	0.06	0.59	NS	0.000
**R SICI, ISI 1 ms**	0.4	0.31	0.6	0.47	-1.38	NS	-0.502
**R SICI, ISI 3 ms**	0.5	0.27	0.9	0.72	-2.13	0.044	-0.736
**R SICI, ISI 5 ms**	1.1	0.53	1.3	0.65	-0.76	NS	-0.337
**R ICF, ISI 7 ms**	1.4	0.74	1.6	0.62	-0.58	NS	-0.293
**R ICF, ISI 10 ms**	1.7	0.61	1.9	0.41	-0.97	NS	-0.385
**R ICF, ISI 15 ms**	1.7	0.69	1.7	0.49	0.00	NS	0.000
**SAI, ISI 2 ms**	71.8	25.63	46.0	11.31	1.31	NS	1.302
**SAI, ISI 8 ms**	76.0	13.32	59.5	14.85	1.45	NS	1.170

(in alphabetical order) CMCT = central motor conduction time; CMCT-F = central motor conduction time calculated through the F wave technique; F = F wave; CSP = contralateral cortical silent period; ICF = intracortical facilitation; ISI = interstimulus interval; L = left hemisphere (right first dorsal interosseous muscle); Lat = latency; MEP = motor evoked potential; NS = not significant; R = right hemisphere (left first dorsal interosseous muscle); rMT = resting motor threshold; SAI = short-latency afferent inhibition; SICI = short-interval intracortical inhibition; bold numbers = statistically significant *p* values.

The general regression model applied to control for the concomitant effect of demographic and anthropometric features, confirmed the significant effect of sex on left and right MEP latency (F = 5.57, *p* < 0.03 and F = 5.38, *p* < 0.03, respectively), longer in males. The same analysis, used by taking into account the cognitive and functional scales, confirmed the statistically significant effect of sex for left MEP latency (F = 8.35, *p* < 0.01), as well for as CMCT-F from left hemisphere (F = 5.41, *p* < 0.03, longer in males), and for SICI at ISI 3 ms from right hemisphere (F = 5.97, *p* < 0.03, lower inhibition in males).

**Fig 1:**
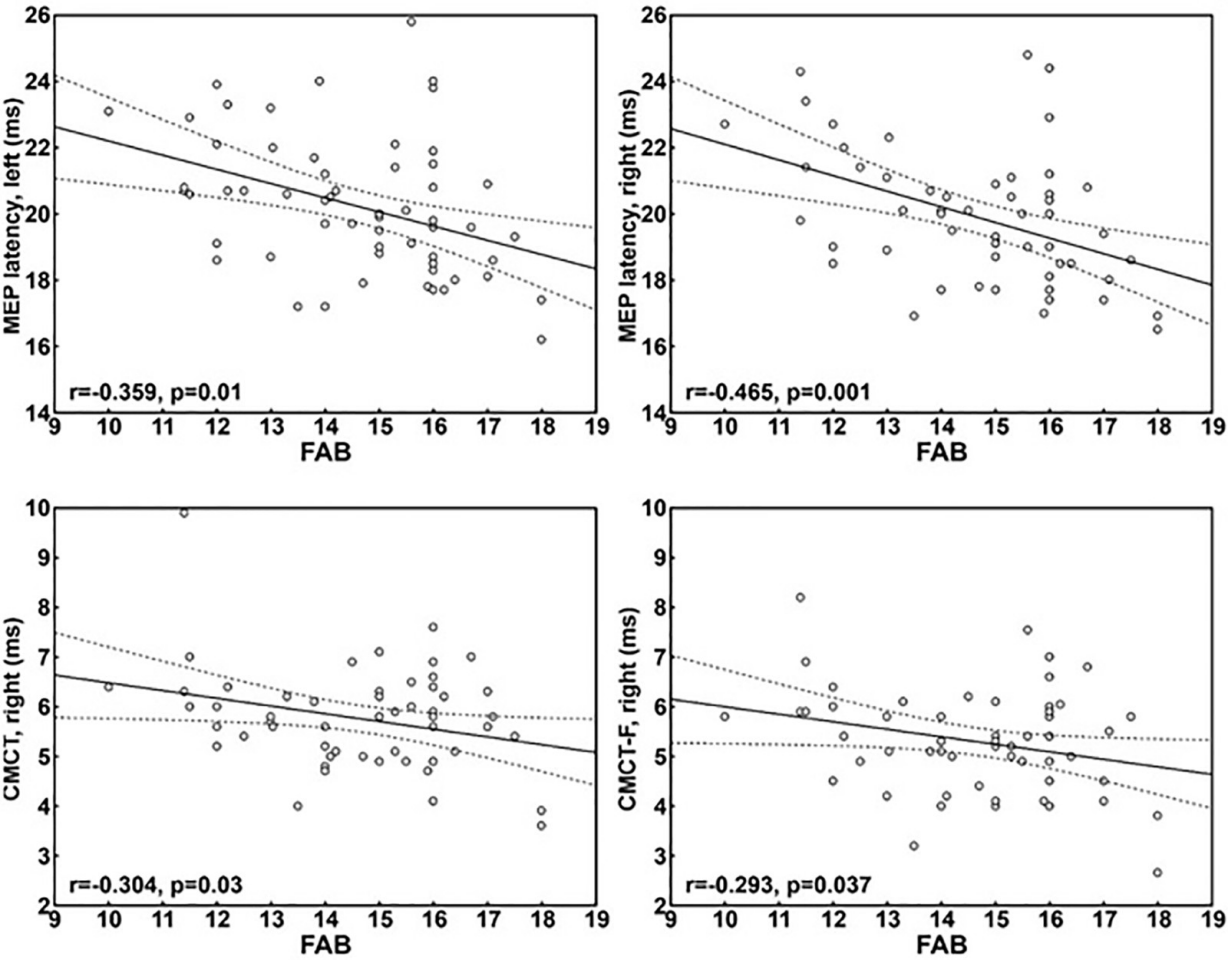
Correlation between FAB scores and TMS data. MEP = motor evoked potentials; CMCT = central motor conduction time; CMCT-F = central motor conduction time calculated through the F wave technique.

Finally, the correlations between FAB and TMS disclosed additional findings (Fig 1): MEP latency bilaterally, and both CMCT and CMCT-F from the right hemisphere inversely correlated with executive functioning, with moderate-to-large correlation coefficients. No significant correlation was found between TMS measures and vascular burden.

## Discussion

### Main findings and proposed pathomechanisms

First, we confirm that males were more likely to have global cognitive impairment (MMSE and MoCA), especially in executive functioning (FAB), as well as more disability (ADL, IADL). Second, males demonstrated delayed motor conductivity (MEP latency and CMCT) respect to females. Third, at paired-pulse TMS level (SICI, ICF) and sensory-motor interaction (SAI), male subjects exhibited a significant disinhibition of the motor responses from the right hemisphere compared to females. Of note, all these changes remained statistically significant after correction for anthropometric and demographic features, except for sex. Finally, diabetes, MEP latency bilaterally, and both CMCT and CMCT-F from the right hemisphere inversely correlated with executive functioning, whereas no correlation was found between TMS and vascular burden.

Preliminarily, it should be acknowledged that there are difficulties in comparing our findings with current literature. Most studies, indeed, did not report their results “by sex” but just “adjusted for sex”. In addition, most studies did not select for cognitive complaints but were population-based, or patients were selected on the presence of various types of vascular damage [64]. Moreover, to date, only few studies have specifically addressed sex-related differences in TMS parameters. Recently, whether cortical excitability to TMS was associated with cognition in healthy individuals and whether sex and education had an impact on this relationship was assessed. The authors demonstrated that lower cortical excitability was associated with better global cognition in healthy females, but not in males [65]. At the paired-pulse TMS level, GABA-mediated intracortical inhibition was found to be greater in healthy females, but it did not significantly change with age [66]. Also, SAI was not different between healthy males and females [67, 68]. Overall, it can be concluded that normal subjects exhibit no significant difference between left and right hemisphere responses in either males or females [69]. Likewise, there is no significant difference between sexes considering each hemisphere separately, as well as between right-handed and non-right-handed individuals for left or right hemispheric responses, in either males or females or for both sexes combined [70]. Nevertheless, in a large cohort of neurologically intact subjects, an independent effect of sex on MEP cortical latency and peripheral motor conduction time at the four limbs was observed, with females showing smaller values than males [71]. Moreover, inter-subject variance and evidence of cortical excitability modulation by other factors, such as stress level, menstrual cycle phase, and reproductive hormonal changes, suggest the presence of other potential factors that may contribute to some variance among healthy individuals [72, 73]. Even fewer TMS studies on sex differences are available in patients with neurological disorders. A recent study has shown that female patients with Parkinson’s disease had a more favorable TMS profile, possibly reflecting a more adaptive cortical compensation or delayed maladaptive changes in the sensory-motor cortex [74]. Conversely, sex did not seem to exert any direct influence on the mechanisms underlying cortical inhibitory deficits in Huntington’s disease [75].

Clinically, we confirmed that diabetes was more commonly reported in males than in females [76]. More interestingly, however, the presence of diabetes negatively correlated with MEP latency, bilaterally. The mechanisms by which diabetes may increase VCI and risk for dementia include increased oxidative stress, cerebrovascular remodeling, endothelial dysfunction/impaired vasodilatation, and altered coagulation [76], eventually leading to deficits in blood flow, including cortical motor areas and cortico-spinal pathways.

Cognitively, our findings are in line with sex differences in the performance on neuropsychological tests, as also seen in AD [77, 78]. Of note, performance on cognitive screening tests, such as MMSE and MoCA, are influenced by the level of education (comparable between males and females), but not by sex [79]. Moreover, it is known that manifestations of vascular brain injury in patients with cognitive complaints can differ by sex. In the present study, there was no interaction between sex and cerebrovascular burden, warranting further studies to explain the sex differences in the pattern of lesion load and/or location. Nevertheless, since neurophysiological mechanisms often precede structural change, this might provide additional insights on the mechanisms involved in brain damage and cognitive decline in patients with VCI.

The localization of vascular lesions might have also contributed to the finding of bilateral increase of MEP latency, i.e., lesions could have hit more or less “strategic” locations belonging to different networks, with different impact on TMS measures. Moreover, microstructural damage of the so-called “normal-appearing white matter” (NAWH) in subcortical ischemic VaD has been recently associated with worse MoCA scores [80]. In this scenario, the clinical consequence of capillary damage may be evident not only in the WMHs, but also in the NAWH, as well as in the “disconnected” (due to vascular injury) cerebral cortex [81]. All these changes might be more prone to occur in male patients, who are more likely to have cognitive impairment (especially in the executive functioning) and disability, thus increasing the overall risk for progression in VaD.

Regarding laterality, research has largely shown that hemispheric specialization and their cognitive abilities differ between sexes, with females demonstrating a more symmetrical brain activity and males a stronger lateralization [82] when performing the same cognitive tasks [83]. This is in agreement with our findings, as a reduced SICI from right hemisphere was noted in males but not in females, although the exact meaning as a predictor of cognitive decline and its role as prognostic marker requires further elucidation.

Physiologically, although normative data on paired-pulse TMS are very few, SICI and ICF in older individuals should not differ between sex or hemispheres, although in subjects >50 years old (as in the present study) smaller intracortical inhibition was observed [84]. A previous study on the neurophysiological correlates of aging-related muscle weakness may possibly explain this result: a lower intracortical inhibition might reflect a compensation for a reduced cortico-spinal excitability, allowing weaker older adults to spread activity in the M1 in the attempt to better sustain motor output [85]. In this context, subjects at high risk for developing VaD, as already proved in those with AD, demonstrated greater brain structure asymmetry, likely due to an impaired metabolism in the hemisphere potentially undergoing greater pathophysiology, GABA-mediated disinhibition, and vulnerability to neurodegeneration [86].

Although limited, both functional MRI and diffuse tensor imaging (DTI) in VCI seem to support this hypothesis [87, 88]. Changes in resting-state patterns of neuronal activity in patients with mild VCI have been reported [87] and probably caused by subcortical WMHs that directly or indirectly impair fiber tract connectivity across cortical and subcortical regions, eventually resulting in hypoperfusion from small vessel disease. The authors hypothesized a compensatory recruitment and plasticity mechanism [87], as already demonstrated in VCI [26, 45], which might be also invoked to explain the delayed motor conduction time observed from the left hemisphere of our patients and the disinhibition of the motor responses from the contralateral side [89]. Finally, in a previous DTI study comparing patients with mild cognitive impairment, mild VCI, and controls [88], although all participants showed decreased fractional anisotropy (FA) and increased mean diffusivity in all cerebral regions except for the occipital areas, those with mild VCI had a greater FA decrease in the parietal region, bilaterally, and in the centrum semiovale than the other groups. Of note, the same patients showed impairment of the frontal executive functions, as also observed in our sample.

Lastly, we previously observed that non-demented elderly patients with ischemic WMHs and clinical features of VCI showed abnormal executive control function. Compared to age- and sex-matched healthy controls, these subjects (considered as a whole group of males and females) did not show any difference for rMT, CSP, or between the two hemispheres, but a significant enhancement of ICF, thus providing a first evidence of functional changes in intracortical excitatory neuronal circuits in mild VCI [90]. After a follow-up period of approximately two years, the same patients showed a decrease of the median rMT and a significant worsening of executive functioning, although without substantial functional impairment. The decrease of rMT during the progression of mild VCI was considered as an index of motor cortex plasticity and viewed as a compensatory mechanism for the loss of motor cortex neurons [91].

### Limitations and perspectives

First, although participants may not be representative of all patients with VCI, they were very homogeneous in terms of demographic and clinical features. Second, the current study did not include healthy controls and, third, as a cross-sectional design, it did not observe TMS measures longitudinally nor changes as a result of an intervention; therefore, causality cannot be inferred from the present findings. Additionally, although MMSE and MoCA are accurate indicators of global cognitive functioning, they remain screening tools. Also, there are no data on sex-specific risk factors for dementia and cerebrovascular disease, such as menopause and erectile dysfunction, since hormonal status has been shown to correlate to both dementia and stroke risk [92].

Finally, the fact that all participants were right-handed might have played a role on the excitability difference observed for SICI from the right hemisphere in males only. It is known indeed that human handedness, and in particular the consistency of the manual preference with age [93], is associated with lateralized differences in the TMS excitability of motor system projections. This possibly reflects physiological differences in cortico-spinal tract function or asymmetry in motor cortical representation [94, 95], although right- and left-handers did not differ in the extent of overlap between muscle representations [96]. More recently, although rMT, MEP amplitude, and MEP latency did not reveal any asymmetry in functional cortical excitability, a significant shift of MEP distribution towards the dominant side was noted [97]. This suggests that laterality may manifest at both level of cortical representation and muscle recruitment, possibly leading to a more pronounced movement on the dominant hemisphere, compared to the non-dominant side, in right-handers [97]. More recently, the comparison of the two cerebral hemispheres in inhibitory processes acting during movement preparation revealed that, while the recruitment of preparatory inhibitory mechanisms is similar within the two hemispheres, the left M1 was more excitable than the right M1 [98].

Lastly, the medications taken by the patients for treating their vascular risk factors do not currently have supporting data with respect to a possible influence on motor excitability parameters [32, 99]. Regarding anti-diabetic drugs, motor cortex excitability is unaffected in insulin-dependent diabetic patients when compared with normo- and hyperglycemic subjects [100], except for a single article reporting a lack of facilitation at ISI of 30 ms in diabetics compared to controls (ISI not tested in the present study) [101].

Future studies should consider all these issues, such as the levels of sex-related hormones, given their potential impact on TMS [102], as well as the possible aging-related MEP differences between sexes [71].

## Conclusions

We confirm the worse cognitive profile and functional status of males with mild VCI compared to females and first highlight sex-specific changes in intracortical and cortico-spinal excitability to multimodal TMS in this population. This points to some TMS measures as potential markers of cognitive impairment, as well as targets for new drugs and neuromodulation therapies.
